# Dental Education

**Published:** 1876-05

**Authors:** W. C. Barrett


### ARTICLE III.

### *Dental Education.*

BY W. C. BARRETT, D. D. S.

The question of what shall be the future status of den tistry, cannot well be solved till we first determine a point of departure, and with some degree of unanimity mark out a prospective course. Shall it like surgery become an adjunct to, or department in general medicine, or shall it aim at a separate existence, as a learned profession ? There is one reason why the dentist who looks to the future of his profession might desire that it be attached to medicine, and that is, that in no other way can it attain to any share of that hoary antiquity which assures a firm standing. Medicine, Law and Divinity have always existed as professions. We are of a later growth, and our vanity might make us desire to claim a little of that respectability which age gives to the other professions. But I cannot see that aside from the gratification of that passion, anything is to be gained by the attempt to shelter ourselves beneath the shadow of their protecting ægis. Certain it is, that medicine will never welcome us until we come through the only door which they recognize,—that of the full medical course and the possession of their diploma. They will justly consider us as intruders and interlopers if we attempt to enter the sacred enclosures of their jealously guarded immunities by any other than the door through which they themselves passed. How then can we as a body, attempt to thrust ourselves upon them without a sacrifice of all dignity on our part?

Taking into consideration the present status of dentistry, pray what right have we to challenge recognition, either as a separate profession or as a particularity in medicine? The latter claim, we are debarred from, for how can we spread and grow as a branch, till we have been grafted into the stock of the parent tree; how can we be specialists in medicine till we first become medical men. We may practice a specialty, but not a specialty *in medicine*, till we shall first have established it as a pre-requisite that every dentist shall pursue a full medical course. We have farmers among us, but that does not make of us a body of agriculturalists.

The great embarrassment is that we have no distinguishing mark by which a dentist may be known from a pretender. All other professions discriminate in this matter, and we shall never be enabled to take rank with them till some indispensible mark or sign of qualification be everywhere recognized and adopted. Medicine has its diploma, and among physicians no man is recognized or fellowshipped unless he possesses this distinguishing evidence. Law acknowledges as proofs of worth, the possession of a diploma, and the admission to practice by competent legal authority. Divinity avows only the formal setting apart and ceremonious induction into their sacred precincts. But what have we to represent these safeguards to privileges, carefully guarded against the uninitiated. We might as well look the matter squarely in the face. A correct diagnosis is necessary to the properly prescribing of the right remedy.

There is no barrier erected in dentistry to separate or distinguish the competent from the incompetent. There is no point at which as in the other profession the students drops his novitiate and assumes the toga of professional manhood; there is no door opened through which the tyro *must* pass to be received as a professional brother. Any man who chooses to drop the pegging-awl or the yard stick, may enter at any point, and straightway assume the professional "Doctor." He need not spend thirty days in preliminary study to be fellowshipped by the great majority of dentists. I, perhaps, write to you that I am a dentist; and what can you do other than to take me at my own estimate of myself? If I claimed to be a physician, you might demand to know at what college I graduated. If I said that I was a lawyer, you could ask me when and where I was admitted to practice; while if I pretended to be a clergyman, you could require knowledge as to how, and when I was ordained as such. But claiming to be a *dentist*, you cannot demand any easily attainable proof of my assertion, nor can I, if I so desire, readily furnish it; for there is no universally recognized standard of qualification. If I am able to show any diploma of D. D. S., it may give you additional confidence in me; but you cannot *demand* this evidence of my standing, because it is not recognized as an indispensable prerequisite to fraternization. You cannot ask of me if I have been regularly admitted to practice, for in but three or four States, is there any such formal admission. You must then on my own recommendation of myself, either give me the right-hand of fellowship, and thereby possibly encourage a mountebank, or you must take the risk of insulting a worthy practitioner. And yet with no line of demarcation between the professional and the unprofessional, we demand admission among the inheritors of the immunities of centuries. Need we wonder that they should spurn connection with a class of men who have no recognized passport to their regard;—that they whose standard of professional ethics is so high, should decline to admit to their altars men who have no means of proving their claim to the honor? What prudent man takes a stranger to his fireside without having first received convincing evidence of his worthiness?

In calling these things to your mind, I am not underrating that calling which I love, nor casting aspersions upon the associates, whom of all men I most respect and serve. I am simply discussing the aspect of acknowledged perplexities, over which I have brooded for many a day, and endeavoring with the light I have, to bring to the relief what, I hope, may prove a remedy. * * * *

What then must we do to secure a proper recognition of our claims to the position to which we believe ourselves entitled? Those who have gone through the curriculum of the school, are within the pale. But such comprise only a small part of dentistry. We cannot force all at once to enter a dental college, and pursue their studies till graduation. What shall we do with the great aggregation, which now calls itself *The Profession?* How shall we establish boundaries to that portion of the broad field of general science, which ought to be exclusively in our possession? I see no other way than through State Legislation. Let the good men in each State meet together and prepare a non-proscriptive law, which shall not be oppressive to those already in practice, but which shall set up some barrier to mark the confines of dentistry. Let the enactment prescribe who shall be considered as worthy, and provide for and establish some duly qualified board, who may examine applicants already in practice, or who propose to enter dentistry, and if found worthy, let that board be empowered to grant some distinguishing diploma. This is what has been done in the State of New York, and its benefits are daily reaped, especially by qualified country practitioners. It provides a practical method whereby those who are now practitioners, but who are unable from family, or other reason, to attend lectures at a college, may become regular in their practice. That it in no way interferes with the regular diploma of the college is abundantly proved by experience. It is rather a benefit to them inasmuch as the diploma of the Board of States Censors is not intended to be an evidence of scholastic training, while it gives ample proof of the standing of its possessor. Every State should enact a law which should recognize as qualified, only such as should have complied with its provisions, or who may be graduates of some reputable college. It need not legislate *against* those who decline or are unable to comply, but it may constructively brand them as quacks, and perhaps refuse them facilities. for the collection of professional fees. But it should draw a clear line of demarcation between the qualified and the unqualified, and grant some special privileges to the former. Too stringent legislation is liable to defeat itself by making the law odious, and arousing an opposition dangerous from mere aggregation of numbers.

This done, we as a profession should absolutely refuse in any way to recognize the practitioner who is not (as in medicine) the graduate of a college, or who has not (as in law,) been regularly admitted to practice by a competent legal board. In time, we should then have a recognized standard, and should be enabled to discriminate between the competent and the incompetent ;—to know who are the mocks and pettifoggers of dentistry. We have schools, and a literature, and learned men—students in science—worthy to honor any profession. Let us have a profession to honor.

There has been much animadversion and stricture of dental colleges, but the fault is not with them. They cannot make bricks without straw. The stream cannot rise higher than the fountain. It is unjust for us to expect them to raise themselves and elevate us too, when we are busy sapping their foundations. When we exalt the general standard of professional attainments, the colleges will rise to the exigencies of the occasion. But it is impossible for them to stop the leaks, when for every opening which they close, the profession itself makes two more. For every qualified man whom they admit, a dozen of the viriest quacks are permitted to push themselves in, and are straightway given the right-hand of fellowship, and the honorary title of "Doctor" is extended to them, till we have made that word of almost as evil repute as Shakespeare said the word accommodate had attained in his day; and done it too by about the same means. Our colleges have opened a reputable door into dentistry, but we scarcely attempt to make students come through it. We recognize every one who strays through the gaps as fully the equal of the D. D. S. Perhaps in some case they are so; but we should have some uniformity, and there is but one State that has provided fully for the proper recognition of the status of such exceptional cases. When dentistry shall regard as a "thief and a robber," he who comes not in by the DOOR, and thus properly sustain the colleges, we will have no more reason to complain of them, than medicine now has to find fault with hers. Pray, what encouragement has a student for entering a college, while fully aware that it will scarce at all tend to raise his professional status? We might as well cease talking of the mote which is in our brother's eye, and begin to pay some attention to the beam that obstructs our own vision.

"The fault, dear Brutus, is not in our stars, but in ourselves that we are underlings." * * * *

